# Coupling effect of nodes popularity and similarity on social network persistence

**DOI:** 10.1038/srep42956

**Published:** 2017-02-21

**Authors:** Xiaogang Jin, Cheng Jin, Jiaxuan Huang, Yong Min

**Affiliations:** 1 Institute of Artificial Intelligence, College of Computer Science, Zhejiang University, Hangzhou, 310027, China; 2 College of Computer Science, Zhejiang University of Technology, Hangzhou, 310023, China

## Abstract

Network robustness represents the ability of networks to withstand failures and perturbations. In social networks, maintenance of individual activities, also called persistence, is significant towards understanding robustness. Previous works usually consider persistence on pre-generated network structures; while in social networks, the network structure is growing with the cascading inactivity of existed individuals. Here, we address this challenge through analysis for nodes under a coevolution model, which characterizes individual activity changes under three network growth modes: following the descending order of nodes’ popularity, similarity or uniform random. We show that when nodes possess high spontaneous activities, a popularity-first growth mode obtains highly persistent networks; otherwise, with low spontaneous activities, a similarity-first mode does better. Moreover, a compound growth mode, with the consecutive joining of similar nodes in a short period and mixing a few high popularity nodes, obtains the highest persistence. Therefore, nodes similarity is essential for persistent social networks, while properly coupling popularity with similarity further optimizes the persistence. This demonstrates the evolution of nodes activity not only depends on network topology, but also their connective typology.

Network robustness is one of the core issues in network science^1^,^2^,^3^,^4^. Early research mainly focuses on static robustness, i.e. the resilience of network connectivity to random errors or targeted attacks of components like nodes or edges^5^,^6^,^7^. Furthermore, dynamics robustness or network persistence, which concerns about the ability of a network to maintain certain states or functions, received major attention^8^. For example, in high voltage networks, cascading failure is a common effect, where a single point of failure on a fully loaded or slightly overloaded system results in a sudden spike across nearly all nodes of the system^9^,^10^. In food-webs, extinction of a certain tropic species may cause a threat to the balance of the food-web, and the persistence of food-webs can be measured as the fraction of initial species remaining at the end of a perturbation^11^,^12^,^13^,^14^. Similarly, in social networks, maintaining individual activity is a key issue towards the overall welfare of the community^15^.

The real social networks possess three particular features, each of which may affect persistence of individual activity. Firstly, diverse network growth modes. Social networks may grow under various modes like following celebrities^16^,^17^, sharing the same hobbies^18^,^19^ or uniformly random ways. Attraction comes from nodes popularity or similarity^20^, and the heterogeneous node and link types may change network persistence even under the same network structure^21^,^22^. Secondly, the cascading effects, i.e. inactivation of a node may cause its neighbors turn inactive. Concepts like *k-*core and *k-*core decomposition are adopted to understand the function of network structure on cascading effects in social networks^23^,^24^,^25^; besides, models initially describing cascading failures^26^ in power grids^7^,^9^,^27^ or epidemic spreading^28^,^29^ are reformed to understand dynamic cascading processes in social networks, including rumor cascades^30^,^31^, influence maximization^32^,^33^,^34^, viral marketing^35^,^36^, etc. Thirdly, unlike power grids or stable food webs, the total number of nodes in social networks is in growth and the node state is also changing^37^,^38^; however, the effect of the coevolution process brings to network persistence remains unclear. Therefore, social network persistence is an interesting and open issue.

Here, based on the three features in social networks, we integrate Papadopoulos’s^20^ network growth model and *k-*core^39^ based cascading processes, which can characterize the coevolution of network growth with diverse modes and the cascading process of node states. To display multiple growth modes of social networks, the extended model contains four steps (See Fig. 1). (1) generate *n* nodes under polar coordinates each with two properties, the popularity of nodes (polar radius 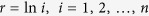) and the similarity with other nodes (random polar angle 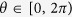). (2) Initially, the network is empty and the adding order of nodes is defined, which corresponds to the descending order of nodes’ popularity or nodes’ similarity, or is uniformly random (in the following, we call them popularity-first, similarity-first and random network growth modes). (3) at time *t*, a node *v* in order *t* is picked up and joins the network. (4) Node *v* connects to a subset of existing nodes *S*, consisting of nodes with hyperbolic distance 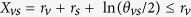, which co-consider the pair nodes’ popularity and similarity with the central angle 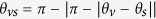, and *v* should connect to no more than *m* existing nodes that obtain the smallest *X*_*vs*_. Here, high popularity stands for small *r* and high similarity means a small angle to 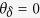. In this way, we inherit the connection rule introduced by Papadopoulos^20^, that is connections link to nodes with small hyperbolic distance, but we split the binding between node popularity and node join time. The generated network still follows the preferential attachment mechanism, but the network growth modes can be very flexible.

To describe the coevolution of state cascading with the network growth, we introduce a cascading process inspired from the definition of *k-*core. When joining in the network, each node has an initial setup of spontaneous activity, say *h*, which is node’s ability to maintain active unconditionally; after the spontaneous time, for each time step, nodes with at least *c* active neighbors could keep active. Otherwise, they would turn inactive permanently. For the demonstration in Fig. 1, *h* is the length of time steps for nodes to remain unconditional active. For the following results, for simplicity, *h* is a relative value of a total number of nodes and *h* ∈ (0%, 100%]. The idea of active neighbor threshold *c* dates back to *k-*core or *k-*degenerate graph in graph theory. The network evolves until all nodes are added, and states for each node never changes, and the persistence of complex social networks can be quantified by the final active ratio of nodes (FAR). Since active nodes satisfy active neighbor threshold *c*, FAR can be seen as a variant measure of *k*-core in dynamic processes in which *k* = *c*.

We analyze the response of the proportion of durable, active nodes with different network growth modes, and further study the coupling effects of popularity and similarity on the promotion of network persistence. The coupling effect in this work means by a combined consideration of nodes popularity and similarity; the network may obtain an enhanced or different persistence. Since the single growth modes (popularity-first or similarity-first) based on one dimension of attraction reveal unique characters, we introduce a weighted score *S*_*i*_ for nodes popularity and similarity to combine the two preferential growth modes together to find whether an optimized mixing growth mode exists (See Eq. 1). These combined growth modes can regulate the joining order of nodes by harmonizing the weights of nodes popularity and similarity. Thus we call this the coupling effect.


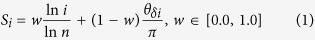


In the weighted score, *w* is the weight parameter, treated as the proportion of node popularity; *n* is the total number of nodes; 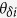 is the central angle of node 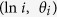 to 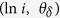 (here we fix 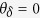). By changing the mixing ratio *w*, we can regulate the coupling effects of nodes popularity and similarity and alter the network persistence under compound growth modes.

## Results

**Figure 2:**
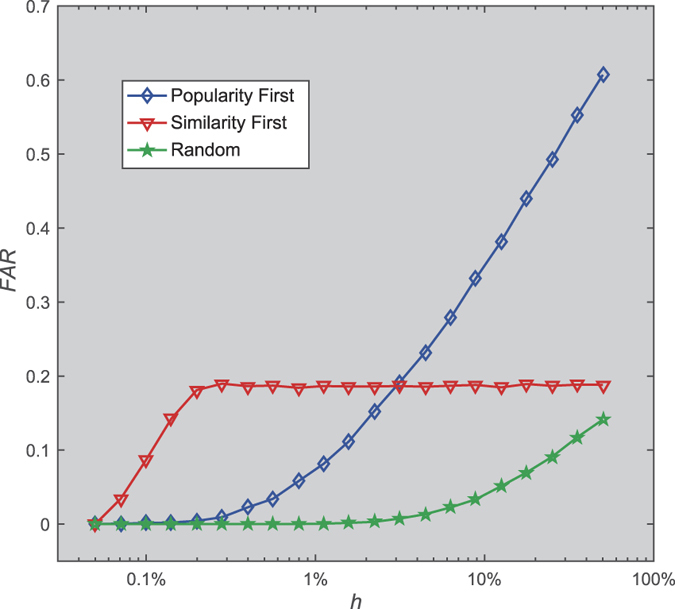
Effects of spontaneous activity *h* on the FARs. The response of FAR to the spontaneous activity for the three growth modes. The blue line represents the popularity-first mode, the red line represents the similarity-first mode, and the green line represents the random mode. Here, *m = *10, *n = *10000, *c* = 60% *m* and *h *∈ (0%, 50%].

As Fig. 2 shows, all the growth modes require a small threshold of spontaneous activities to keep FAR > 0, and the exact value is related to the specific modes. Throughout the whole *h*, random mode remains the poorest in maintaining network activities, with a FAR staying in low values. The similarity-first mode is robust throughout *h*, the initial FAR quickly grows with the increase of *h* to a point and then keeps unchanged forever. The Popularity-first mode enlarges FARs as *h* grows. Therefore, there is an intersection point where when *h* increases to a critical value, popularity-first mode reaches the same FAR as the similarity-first mode, which is the unchanged maximum FAR of the similarity-first mode. When *h* keeps growing, the popularity-first mode replaces similarity-first mode and continuously enlarges FAR.

**Figure 3:**
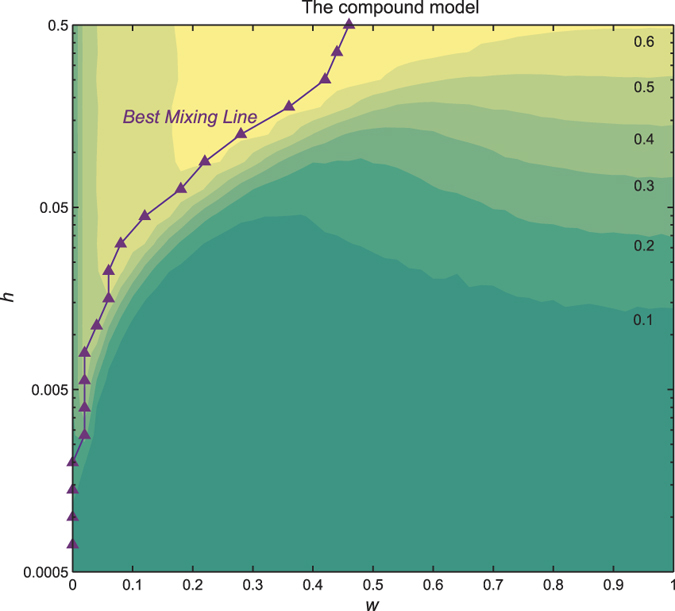
The response of the FAR to the mixing ratio *w* and spontaneous activity *h*. Here, *w* ∈ [0,1.0], *h *∈ [0.5%, 50%], *m* = 10, *n* = 10000 and *c* = 0.6 *m. w* is the proportion of the weight of node popularity. *w* = 0 and 1 give growth modes equivalent to the similarity-first and popularity-first growth modes, respectively.

By considering coupling of popularity and similarity (See Fig. 3), as *w* increases, the FAR first increases rapidly to a peak value, then drops rapidly to a minimum value that can be as bad as the simple similarity-first or popularity-first growth mode (when *w* = 0 or 1). With an appropriate mixing rate, the FAR of the compound growth mode is at least double the size of a simple similarity-first or popularity-first growth mode. The peak value of the FAR always emerges at *w *< 0.5. This result suggests that adopting similarity-first growth mode as a base, and giving moderate priority to nodes with high popularity to join in can significantly improve network persistence. It means that, to some extent, when regulating network persistence, similarity functions as the basic elements, while nodes with high popularity are the amplifiers.

## Discussion

### Popularity-first vs similarity-first growth modes

**Figure 4:**
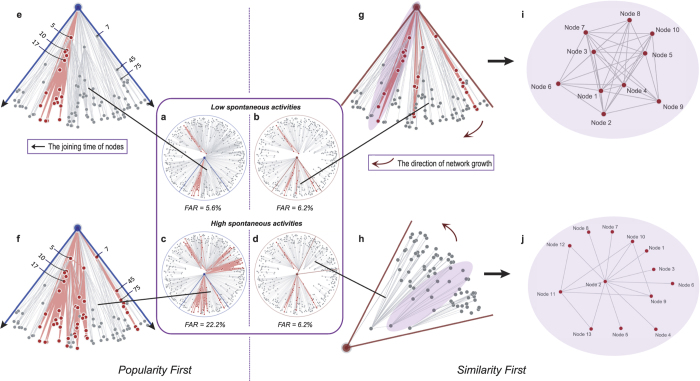
Final static structure visualization analysis for popularity-first and similarity-first growth modes. (**a**) Final slice for a popularity-first mode with low spontaneous activity *h* = 8%. (**b**) Final slice for a similarity-first mode with low spontaneous activity *h* = 8%. (**c**) Final slice for a popularity-first mode with high spontaneous activity *h* = 30%. (**d**) Final slice for a popularity-first mode with high spontaneous activity *h* = 30%. (**e**) Zoom in a sector for (**a**). (**f**) Zoom in a sector for (**c**). (**g**) Zoom in a sector for (**b**) (**h**) Zoom in a sector for (**d**). (**i**) The topology of a specific part in (**g**). (**j**) The topology of a specific part in (**h**). Here, *n* = 2000.

To discover the mechanisms behind popularity-first and similarity-first growth modes, we do data visualizations for the final states of coevolving networks. Our visualization analysis contains both growth modes under low spontaneous activity and high spontaneous activity, which considers both sides of the interaction point (See Fig. 4a–d). The node sequence here is generated once, and only the growth mode or spontaneous activity matters.

In the popularity-first mode, nodes are added as the radius increases (node popularity decreases). Since existing nodes are nodes with the highest popularity, similar high popularity nodes can have sufficient connection with each other and lead to some structure rich in triangles (triadic-closure). As polar radius here is bonded as the log of joining time, a small radius difference can result in enormous gaps. We pick up three nodes for each of the two subgraphs; the left subgraph contains nodes continuously joined within a short time interval, and nodes in the right subgraph are alienated on joining time (See Fig. 4e). For the left subgraph, nodes connect mutually with each other and maintain activities, which leads to the persistence of the subgraph. For the right subgraph, though it contains high popularity nodes, the period for joining is very long. Under low spontaneous activity, the early joined nodes already turn inactive due to lack of active neighbors before the later nodes join. As a cascading failure, the later joined nodes turn inactive as well. Unlike the low spontaneous activity, when *h* increase (see Fig. 4f), the early joined nodes with high popularity can remain active when the later nodes join, and the cascading effects keep more members within the subgraph keep active. This amplification of activities can also be seen in the left subgraph maintaining a larger amount of active nodes. This shows that networks under the popularity-first mode contain dense links, thus forming durable, active groups are largely dependent on *h* and the joining interval of nodes. This graph also shows that structural characteristics such as node degrees or the great connected component show limitations when evaluating the final individual activities^23^,^25^.

Unlike the popularity-first mode, the similarity-first mode is robust throughout *h*. As Fig. 4g,h shows, the group can be survived by first adding a node with high popularity, continuous with a group of very similar nodes who almost at the same polar angle and sufficiently connect with each other. Otherwise, the followed nodes would not share high clustering coefficients. That is, when links within the group are mainly focused on a single high popularity node and only a few links are between other nodes and the group is sure to turn inactive (see Fig. 4i,j). This tree-like topology cannot keep nodes active; whether we increase *h* or not, the nodes would turn inactive finally. However, for the highly connected part, since their joining time is within a short-time internal, only a small *h* is enough to keep the group alive.

The similarity-first growth mode restricts the entry of nodes from a specific type, and gradually becomes more diverse, it can maintain small active groups but cannot effectively enlarge them or lead to a higher FAR. The popularity-first growth mode controls the entrance of nodes based on descending order of popularity. It relies on the existence of longer spontaneous activity times to avoid cascading failures, and it can enlarge an already active group. By combining a dynamic analysis based on spontaneous time and a static analysis of visualization and network topology, we find that these two growth modes play complementary roles in stabilizing social networks. Therefore, combining the advantages of the two growth modes in a compound growth mode is a reasonable approach; mixing properties of nodes may give an optimal link type on forming high-persistence social networks.

### Coupling effect of the compound model

**Figure 5:**
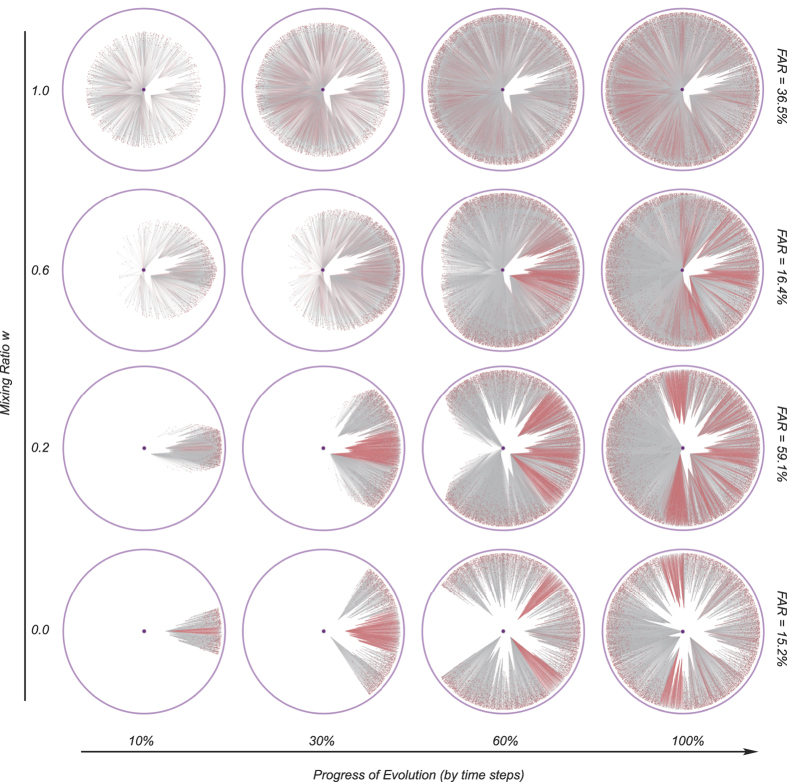
Visualization of the dynamic process under a compound growth mode. Here, each row shows the dynamic process of a particular network about its growth and the expansion of its activity. The four rows correspond to four values of the mixing ratio *w* = 0.0, 0.2, 0.6 and 1.0. Red nodes are active nodes and gray nodes inactive. Red lines between two nodes indicate that both nodes are active. The light blue circle is the margin of the network, and the large purple node is the center of the circle. Here, *m* = 10, *n* = 10000, *c* = 0.6 *m* and *h* = 7%.

To clarify the coupling effect which controls the performance of compound growth mode, we visualize the coevolution of network structure and node states with various mixing ratios. Figure 5 shows four particular dynamic processes corresponding to mixing ratios 0.0, 0.2, 0.6 and 1.0, respectively (with a spontaneous activity of 10%). For *w* = 0.0 and 0.2, the growth of the network exhibits a fan-shaped expansion in hyperbolic space, in which the addition of nodes substantially follows a similarity-first growth mode. For *w* = 1.0, network growing is through radial expansion, in complete accordance with a popularity-first growth mode. Moreover, for *w* = 0.6, the growth (especially at late times) has a transverse wavy pattern. These forms of expansion provide an explanation for the nonlinear behavior of the compound growth mode.

In the similarity-first mode, an active group obtains a high-popularity node added first, and then a batch of very similar nodes to connect sufficiently with each other (See Figs 3 and 5, *w* = 0.0). By weighing a bit on the popularity, the high-popularity node can be added a little earlier, which can survive more nodes which would otherwise turn inactive, and thus the active groups grow bigger (Fig. 5, *w* = 0.2). Meanwhile, when enlarging the weight of node popularity, when *w* = 0.6, the weights for popularity and similarity are very close. In this case, the preferential growth mode degenerates to be nearly the random growth mode. The mixing mode fails to enhance network persistence. The form of network growth in hyperbolic space reveals the fundamental role of similarity in network persistence and explains the critical role of the joining time of high popularity nodes.

Despite the vast and growing literature on network robustness, the importance of social network persistence has not received significant attention and is not fully understood. In this work, we present a model which can describe the persistence of complex social networks. In spite of its simplicity, the model integrates three features of social networks, the cascading effects, the coevolution of network growth and node states changes, and different network growth modes along with the connectivity patterns^40^,^41^,^42^. Our findings expose a critical role of network growth modes in maintaining individual activity and clarify the various roles of nodes popularity and similarity on network persistence. Furthermore, we explore combined growing modes based on the coupling effects of nodes popularity and similarity and find the best mixing ratio and best mixing line that promotes the network to a much higher persistence than any single growth mode.

Interestingly, our work can explain persistence in Weibo (http://weibo.com, like Twitter) and Renren (http://www.renren.com, like Facebook), two famous social network sites in China. Initially, Weibo invited celebrities in all fields to join and attracted fans of celebrities as well^43^,^44^; and Renren focused on university students, especially Freshmen who kept in touch with old classmates and made new friends. Renren adopted similarity based growth mode but no clear popularity based modes^40^,^45^. Inactivation of some users caused massive amounts of users turned inactive and new Freshman lost interest to join, the similarity based growth mode collapsed^46^. In contract, Weibo suffered from user freshness recession once, and it updated strategy with two aspects: mining attractive topics and optimizing experience in topic discussion. This strategy promoted the spontaneous activity of users who concerned about idols or hot issues of society^47^,^48^, and largely enhanced interactions between similar users^19^,^41^. Coupling effects of nodes popularity and similarity, along with the rise of spontaneous activity, led to a better prosperity of Weibo. Its share price outstripped the worldwide social network Twitter in 2016^49^. These two realistic cases consist of our results.

## Methods

### Papadopoulos’s model and the extension

In the original model^20^, each node has two properties, namely popularity and similarity, which correspond to polar radius *r* and polar angle *θ* in polar coordinates; in addition, the hyperbolic distance 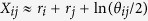 is introduced to characterize the distance between two nodes *v*_*i*_ and *v*_*j*_.

The network grows as follows: (1) initially (*t *= 0), the network is empty; (2) at discrete time step *t*(>0), a node *v*_*t*_ is added to the network with assigned popularity *r*_*t*_ = ln(*t*) and uniformly random generated similarity 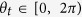; and (3) a new incoming node can connect to *m* existing nodes that have already been added to the network and that have the least hyperbolic distances from it. By iterating the growth process, a complex network with a power-law distribution can be obtained, which is more realistic than preferential attachment.

This model offers a way to generate a growing network based on modified preferential attachment. And we modified this in two parts. First, when each node joins the network, we not only consider building new connections but also changing the individual activity states (active or inactive); Second, we first generate all the nodes, and then change their joining order, so the nodes popularity *r* no longer bonded to the adding time *t*. In all these growth modes, we inherit the node connectivity rule from Papadopoulos’s model by judging the hyperbolic distance. In this way, the network can attract nodes based on elastic node properties like popularity-first, similarity-first, random or the combined properties to build multi-type links and still follow the preferential attachment mechanism.

### The idea of *k-*core

In graph theory, a *k-*degenerate graph (also known as a the *k-*core number, *k-*shell number) is an undirected graph in which every subgraph has a vertex of degree at most *k*: that is, some vertex in the subgraph touches *k* or fewer of the subgraph’s edges^39^. The degeneracy of a graph is the smallest value of *k* for which it is *k-*degenerate. The degeneracy of a graph is a measure of how sparse it is, and is a constant factor of other sparsity measures such as the arboricity of a graph.

The active neighbor threshold *c* we set in this work goes back to the idea of *k-*core in graph theory. We can think the active groups of nodes as the analogy to a c-core subgraph of the original network. One should note that when we analyze on the active groups, the inactive parts of the network is not removed.

## Additional Information

**How to cite this article:** Jin, X. *et al*. Coupling effect of nodes popularity and similarity on social network persistence. *Sci. Rep.* **7**, 42956; doi: 10.1038/srep42956 (2017).

**Publisher's note:** Springer Nature remains neutral with regard to jurisdictional claims in published maps and institutional affiliations.

## Figures and Tables

**Figure 1:**
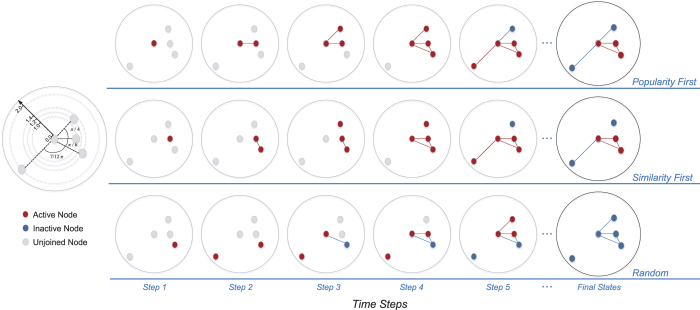
Geometric interpretation of network formation by nodes popularity and similarity, and evolution of individual activity. The process is shown in polar coordinates. At each time step *t*, a node *v*_*i*_ = (*r*_*i*_,*θ*_*i*_) with the active state (red point) is added to the network and can connect to at most *m* existing nodes (here *m* = 2) whose hyperbolic distance from *v*_*i*_ is less than *r*_*i*_ (i.e. within the green areas). If there are *m*′ > *m* existing nodes within the green area of node *v*_*t*_, then *v*_*t*_ preferentially links to the *m* nearest nodes regarding hyperbolic distance. The hyperbolic distance between current node *v*_*i*_ and an existing node *v*_*j*_ is 
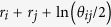
. An active node can remain in the active state unconditionally within *h* time steps (we call this the spontaneous activity) since it was added. After the spontaneous activity, the node remains in the *active* state on the condition that it has at least *c* active neighbours (we call this active neighbour thresholds, e.g. node 1 at time steps *t* = 3 and 4); otherwise, it changes to the *inactive* state (e.g. blue node 2 at time step *t* = 4) and remains in this state indefinitely thereafter. The order in which nodes are added can be freely chosen. In this illustration, we use a demonstration of five nodes to show the network growths and activity changes under popularity-first, similarity-first and random growth modes. We set spontaneous activity *h* = 2 and active neighbour threshold *c = *2 both as absolute values here. But the following discussion adopts the relative value within range [0%, 100%] for simplification, and *h* is a relative value of total number of nodes, *c* is a relative value of *m*.
